# Network analysis of quality of life and depression in a randomized breast cancer trial with ten-years follow-up

**DOI:** 10.1038/s41598-026-51322-3

**Published:** 2026-05-04

**Authors:** Sofia Hampf, Eleni Kolokotroni, Carl Blomqvist, Liisa Hakamies-Blomqvist, Georgios Stamatakos, Riikka Huovinen, Pirkko-Liisa Kellokumpu-Lehtinen, Tiina Saarto, Paula Poikonen-Saksela

**Affiliations:** 1 Faculty of Arts, Psychology and Theology, Åbo Academi University, Turku, Finland; 2 In Silico Oncology and In Silico Medicine Group, Institute of Communication and Computer Systems, School of Electrical and Computer Engineering, National Technical University of Athens, Athens, Greece; 3 Comprehensive Cancer Center, Helsinki University Hospital, University of Helsinki, Helsinki, Finland; 4 Department of Oncology, Turku University Hospital and Faculty of Medicine, University of Turku, Turku, Finland; 5 Faculty of Medicine and Health Technology, Tampere University and Cancer Center, Tampere University Hospital, Tampere, Finland

**Keywords:** Breast cancer survivors, Quality of life, Network analysis, EORTC QLQ‐C30, Depression

## Abstract

Due to the growing amount of breast cancer survivors we must understand their long-term quality of life (QoL) better. Many breast cancer survivors suffer for years from lower functioning capacity and several symptoms, but outcomes regarding their global Quality of Life (gQoL) are less clear. The current study explored the links between functions, symptoms, and gQoL using a network design. We used data from 364 women who had completed adjuvant chemotherapy or started adjuvant endocrine therapy. QoL, symptomatology and functioning were measured by the EORTC QLQ‐C30 and BR‐23 questionnaires, and depression by the Finnish version of Beck’s 13‐item depression scale. The multivariate inter-dependence between the symptom scales and gQoL was analyzed via regularized partial correlation networks (graphical LASSO). These networks identify associations, not directionality or causality. Our aim was to explore how the functions and symptoms associate with each other and with gQoL during ten-year follow-up, and to identify the most important scales in the network using centrality analysis. The results showed that three key areas—fatigue and physical functioning, emotional functioning and depression, and social and role functioning—emerged as most central to gQoL across all time points. Interventions targeting these areas may therefore have the greatest potential to improve long-term QoL in breast cancer survivors.

**Supplementary Information:**

The online version contains supplementary material available at 10.1038/s41598-026-51322-3.

## Introduction

With increasing rate of breast cancer survival, there is a pressing need to better understand the long-term quality of life (QoL) in these survivors^1^. QoL as defined by the WHO is an individual’s perception of their position in life in the context of the culture and value systems in which they live and in relation to their goals, expectations, standards, and concerns^2^. It is therefore a multidimensional concept where both objective life conditions and a person’s subjective appraisals must be considered^3^. Health-related quality of life (HRQoL) usually refers to those aspects of QoL that are influenced by health status, but these two concepts are often used interchangeably^4^. HRQoL can be measured by both generic and disease-specific instruments, and the cancer-specific HRQoL instruments also include an evaluation of side-effects related to the disease itself or its treatments.

### Quality of life in breast cancer survivors

QoL is shown to be most impaired during the active treatment phase including surgery, chemotherapy, targeted therapy, and radiotherapy, due to their side-effects, but it usually starts to improve within the first years after cessation of treatment^5^. However, endocrine treatment is used for even up to ten years and can cause long-term toxicity^6^. Studies show that even five to ten years after the illness, breast cancer survivors experience several adverse health consequences, such as fatigue^5,7–11^, sleep disturbance^5,7,8^, menopausal symptoms^5,8^, and depression^12^. Restrictions in physical^7,9^, cognitive^5,7,10^, social^7,10^, role^9,10^, and emotional functioning^7^ are also frequently reported. Studies focusing on long-term global QoL (gQoL)—a subjective measure of patients’ perceived QoL—have yielded more mixed findings. There is some evidence for impaired QoL^13^, but many studies report that long-term QoL is similar between age-matched controls and breast cancer survivors, despite deficits in functioning and a heavier symptom burden in breast cancer survivors^5,7,14,15^. This has led some researchers to conclude that gQoL clearly is more than the sum of its parts^14^. For a more thorough understanding of long-term QoL and its many aspects, it would be beneficial to also examine how the specific symptoms and functions relate to each other and to QoL, in addition to identifying which restrictions persist. Knowledge about the patterns and key dimensions underlying QoL could help inform future interventions as to where to target their efforts in order to have the greatest impact on the long-term well-being in breast cancer survivors.

### Interconnections between different symptoms and functional impairments

To explore how different aspects of well-being relate to long-term gQoL, in the present study we utilized a network analysis method (graphical LASSO) based on the network theory of mental disorders. Network theory posits that instead of symptoms being caused by certain disorders, it is the direct interactions of the symptoms that give rise to the mental disorder^16^. When we study the quality of life of cancer patients the observed/reported symptoms typically correlate with each other, and it is crucial to understand their interplay to grasp the causal complexity of the QoL outcome. Symptoms of psychopathology are causally connected through different biological, psychological, and societal mechanisms, which form networks that can become self-sustaining, if the connections are sufficiently strong. A vulnerable network is thus one characterized by strong connections between different symptoms, and a network stuck in a disordered state is what gives rise to mental disorder^16^. There are certain differences in applying the Network Theory to QoL as compared to the earlier work on psychopathology. Instead of systems of interacting dysfunctions, the QoL network consists of states (e.g., pain) and functional levels, but also of contexts (e.g., social support) and subjective evaluations (e.g., meaning). That, in addition, many nodes are interpretations of states, rather than states themselves, makes them more context-dependent and reversible. gQoL should be seen as an emergent node, since its relationship to other nodes is bidirectional: how a patient experiences their gQoL can modulate their appraisal of the other nodes.

A previous research report from the BReast cancer and EXercise (BREX) trial showed that fatigue and scales related to emotional health had the strongest associations with gQoL at one year after baseline^17^, but to our knowledge a network analysis of long-term QoL in breast cancer survivors has not been conducted.

Our aim in the present study was to explore the interconnections between gQoL, symptomatology, functioning, and depression at one, three, five and ten-year follow-up in early breast cancer patients in the BREX intervention trial. By investigating the networks formed by the different aspects of QoL at the different time-points, we aimed to gain knowledge about how the networks develop over time, how the functions and symptoms relate to each other and to gQoL, and which factors have the strongest connections with gQoL at different time points. Better knowledge about central factors affecting QoL is vital when planning interventions for patients at different stages in their recovery.

## Methods

### Participants

The BREX study is an open prospective randomized clinical trial exploring various health effects of a one-year intensive aerobic exercise intervention among breast cancer survivors. A total of 573 women aged 35–68 years who had recently completed adjuvant chemotherapy or started endocrine therapy of early breast cancer were recruited in the study between September 2005 and September 2007 from three university hospitals (Helsinki, Tampere, and Turku). Participants over 68 years of age and patients treated with only radiotherapy were excluded from the study to optimize the homogeneity of the study group. Patients unable to participate in training, due to severe cardiac disease or musculoskeletal disorders also were not included. Detailed inclusion and exclusion criteria are presented in previous publications^18,19^. The recruitment rate was 54% of the potentially eligible patients and 31% of all screened patients^18^.

After baseline visits, which included surveying of medical history and a medical examination, patients were randomized into a one-year supervised exercise training and a control group. The effect of this exercise intervention on the QoL after one and five years from randomization has been reported in our previous publication^18,20^. The one-year exercise intervention included both supervised and home training. In the current study the exercise and the control groups are analyzed as one group, since no significant difference in QoL was found between the groups^19^.

In the present study, the follow-up data from the one-, three-, five- and ten-year follow-up visits are used. From the 573 patients initially enrolled, a total of 380 patients participated in the ten-year follow-up visit. After excluding patients who participated only once during the follow-up period and those with missing long-term results (i.e., five- or ten-year follow-up), the final analysis encompassed 364 patients.

### Measures

The clinical investigations were conducted in line with usual follow-up practice. These included basic laboratory safety tests and radiological examinations. During the baseline visit the medical history of the patients was examined and they went through a medical examination and laboratory tests. In addition, the patients filled out a questionnaire covering QoL, basic demographics, and lifestyle issues, and carried out physical performance tests. QoL questionnaires and physical performance tests were repeated after 1, 3, 5 and 10 years.

QoL was measured by the EORTC QLQ-C30 (European Organization for Research and Treatment of Cancer Quality of Life Questionnaire: Core questionnaire) instrument^21^ with the addition of the breast cancer module supplement BR-23 (European Organization for Research and Treatment of Cancer Quality of Life Questionnaire: BReast cancer module^22^;). EORTC QLQ-C30 contains 30 items and consists of nine multi-item scales: five functional scales (physical, role, cognitive, emotional, and social); three symptom scales (fatigue, pain, and nausea and vomiting), and a global health and QoL scale^21^. The Global health/QoL scale (hereafter gQoL) describes patients’ subjective quality of life and is based on two questions about physical condition and overall QoL. In addition, several single-item symptom measures were included for assessing dyspnea, appetite loss, sleep disturbance, constipation, and diarrhea. The breast cancer specific module BR23 includes 23 items to assess the impact of common breast cancer treatment modalities (surgery, chemotherapy, radiotherapy, or endocrine treatment) upon women’s well-being^22^. The Finnish modified version of Beck’s 13-item depression scale (BDI^23^;) was used to assess depressive symptoms. The questionnaires were completed at each follow-up visit.

### Ethical approval and informed consent

The BREX study was conducted in accordance with the Declaration of Helsinki. The study protocol of the trial was approved by the local ethical committee of the Helsinki University Hospital. The patients received oral and written information, and all patients gave written informed consent before entry into the study. All experiments comply with relevant guidelines and regulations. The trial is registered in the Helsinki and Uusimaa Hospital District Clinical Trials Register (www.hus. fi) with the trial number 210590 and at http://www.clinicaltrials.gov/ with the identifier number NCT00639210 and, registration date 20/03/2008.

### Statistical analysis

#### Missing data

Missing data at the scale level stayed under 6.2% across all time points (year one: 5.1%, year three: 2.4%, year five: 6.2%, year ten: 6.0%). The missing values were imputed using a trajectory-based method for longitudinal data, specifically the copyMean function from the R package longitudinalData^24^.

#### Network construction

Networks of partial correlation coefficients were constructed for one-, three-, five-, and ten-year follow-ups. In these networks, nodes represent questionnaire scales, and they are connected by edges representing partial correlation coefficients. These coefficients signify the residual association between two variables once the influences of all other variables have been considered. The selection of the scales included in this study was based on their moderate to high association with gQoL (Spearman correlation from 0.3 to 0.49 were defined as moderate, and ≥ 0.50 as strong^25^) , both at baseline and at 1-year follow-up, as detailed in a previous publication^17^. The variables omitted from further analysis were one-item symptom scales relating to treatment side-effects, as it was considered unlikely that these would increase their importance over time.

The computation of partial correlations followed the procedures outlined in Constatini et al.^26^ and Epskamp et al.^27^, using the R package qgraph^27^. Initially, a zero-order correlation matrix was computed, incorporating Pearson correlations between continuous scales, polyserial correlations between continuous and ordinal scales, and polychoric correlations between ordinal scales. Scales with 7 levels or fewer (i.e., single- and two- item scales) were treated as ordinal, and the rest as continuous^28^. Subsequently, the regularization technique known as graphical LASSO (Least Absolute Shrinkage and Selection Operator)^29^ was applied to estimate partial correlations from the zero-order correlation matrix and to identify and eliminate spurious (i.e., false positive) connections—non-zero partial correlations that are zero in the true underlying network. The sparsity of the resulting network (i.e., the proportion of non-zero connections) was controlled by the tuning parameter λ. The optimal λ was selected from a sequence of candidate values by minimizing the extended Bayesian information criterion (EBIC) (optimal λ values: ~ 0.044, 0.040, 0.037, and 0.051 for the one-, three-, five-, and ten-year follow-ups, respectively). The EBIC tuning hyperparameter, γ, was set to the recommended value 0.5 that avoids most spurious edges^30^. The approach mitigates the loss of statistical power associated with multiple testing corrections, when aiming to control for spurious connections^31^.

Networks were visualized according to Fruchterman-Reingold layout for weighted networks^32^, that aims to place highly connected nodes close to each other. Green and red edges indicate positive and negative partial correlations, respectively. Thicker and more saturated edges indicate stronger partial correlations.

#### Network analysis

The centrality indices of strength, closeness, and betweenness for each node were calculated to assess their importance in the networks^33–36^. Strength reflects the cumulative weights of edges originating from the node. Nodes with high strength exhibit strong direct connectivity with others. Closeness, inversely related to the sum of distances from the node to all others in the network, measures how quickly a node can influence or be influenced by others, through both direct and indirect connections. Betweenness quantifies the number of shortest paths passing through the node, highlighting its role in connecting other nodes.

The stability of each network’s structure and reliability of centrality estimates was assessed using the R package bootnet, focusing on the correlation stability coefficient (CS-coefficient) to measure robustness via case-dropping subset bootstrap. Additionally, bootstrap analyses were conducted to examine edge-weight variability and identify significant differences in edge weights or node strength-centralities across 1000 bootstrapped networks.

The stability of the network structure over time was assessed by employing the permutation-based statistical test implemented in the R package NetworkComparisonTest^37^ and by computing Spearman correlations between the edge weights of the networks.

More details on methods can be found in the Supplement.

## Results

### Patient characteristics

The median age of the patients at baseline was 53 years (range 35–68). One hundred and sixty-three (45%) patients were premenopausal and two hundred and one (55%) postmenopausal. Two hundred and twenty-nine (63%) patients were lymph-node positive. Two hundred and thirty-one (64%) patients had breast-conserving surgery and one hundred and thirty-three (37%) had a mastectomy. Three hundred and twenty-five patients (89%) received chemotherapy and three hundred (82%) received endocrine therapy. The clinical characteristics at baseline of the 364 patients analyzed are presented in Supplement Table 1. QoL and depression at one-, three-, five-, and ten-years follow-up are presented in Table 1.

**Table 1:** Health related quality of life at one, five and ten years measured by the EORTC QLQ-C30 and EORTC QLQ-BR23 and the BDI questionnaires.

Variables, Mean ± SD (range)	One year	Three years	Five years	Ten years
EORTC-QLQ-C30 scales				
Global QoL	75.5 ± 19.0 (0–100)	73.9 ± 19.9 (0–100)	75.5 ± 18.2 (16.7–100)	75.7 ± 19.2 (0–100)
Physical functioning	85.9 ± 14.5 (33.3–100)	86.5 ± 14.4 (26.7–100)	86.1 ± 14.5 (26.7–100)	85.4 ± 15.3 (20–100)
Role functioning	90.0 ± 17.7 (0–100)	90.0 ± 18.8 (0–100)	91.2 ± 17.3 (0–100)	89.8 ± 18.1 (0–100)
Social functioning	94.4 ± 14.8 (0–100)	93.6 ± 16.1 (0–100)	93.9 ± 14.3 (16.7–100)	93.5 ± 16.1 (0–100)
Emotional functioning	84.2 ± 17.0 (16.7–100)	83.4 ± 19.5 (0–100)	84.2 ± 17.5 (0–100)	84.6 ± 18.7 (0–100)
Cognitive functioning	85.5 ± 19.1 (0–100)	85.9 ± 18.8 (16.7–100)	87.1 ± 18.4 (0–100)	86.5 ± 18.0 (0–100)
Fatigue	22.3 ± 19.3 (0–100)	23.5 ± 21.8 (0–100)	21.6 ± 19.8 (0–100)	21.7 ± 19.4 (0–100)
Insomnia	27.6 ± 30.5 (0–100)	28.4 ± 30.3 (0–100)	25.8 ± 27.9 (0–100)	25.8 ± 26.5 (0–100)
Pain	17.2 ± 21.3 (0–100)	18.6 ± 22.0 (0–100)	16.4 ± 20.3 (0–100)	17.2 ± 21.8 (0–100)
Nausea and vomiting	2.3 ± 7.2 (0–66.7)	2.3 ± 8.8 (0–100)	2.0 ± 7.0 (0–66.7)	1.6 ± 7.5 (0–100)
Dyspnea	5.7 ± 16.0 (0–100)	6.2 ± 15.0 (0–66.7)	7.3 ± 17.8 (0–100)	7.8 ± 18.7 (0–100)
Appetite loss	3.1 ± 10.6 (0–66.7)	3.9 ± 14.0 (0–100)	2.3 ± 10.3 (0–66.7)	3.6 ± 13.1 (0–100)
Constipation	11.0 ± 20.0 (0–100)	10.8 ± 18.7 (0–100)	11.8 ± 21.1 (0–100)	11.3 ± 19.8 (0–100)
Diarrhea	5.1 ± 14.2 (0–100)	5.5 ± 14.9 (0–100)	6.1 ± 17.0 (0–100)	5.4 ± 14.9 (0–100)
Financial difficulties	5.4 ± 16.9 (0–100)	4.9 ± 15.3 (0–100)	5.3 ± 18.0 (0–100)	4.7 ± 16.1 (0–100)
EORTC-QLQ_BR23 scales				
Body image	75.6 ± 25.1 (0–100)	78.0 ± 23.7 (0–100)	80.0 ± 21.0 (0–100)	82.9 ± 21.0 (0–100)
Sexual functioning	33.6 ± 26.7 (0–100)	30.5 ± 26.0 (0–100)	29.6 ± 25.7 (0–100)	25.6 ± 24.4 (0–100)
Sexual enjoyment	62.3 ± 25.2 (0–100)	58.5 ± 24.9 (0–100)	63.1 ± 24.6 (0–100)	59.5 ± 25.0 (0–100)
Future perspective	64.6 ± 25.9 (0–100)	69.3 ± 25.8 (0–100)	71 5 ± 25.1 (0–100)	73.8 ± 25.7 (0–100)
Systemic therapy side effects	16.0 ± 12.1 (0–83.3)	15.7 ± 12.4 (0–61.9)	14.1 ± 12.0 (0–66.7)	13.1 ± 11.5 (0–61.9)
Breast symptoms	10.5 ± 12.8 (0–66.7)	8.1 ± 12.2 (0–61.9)	6.5 ± 10.0 (0–50)	6.1 ± 11.8 (0–83.3)
Arm symptoms	17.4 ± 18.3 (0–88.9)	13.5 ± 16.7 (0–100)	12.4 ± 17.2 (0–100)	11.4 ± 17.1 (0–100)
Upset by hair loss	8.1 ± 23.3 (0–100)	4.9 ± 18.0 (0–100)	4.3 ± 15.3 (0–100)	4.5 ± 14.8 (0–100)
BDI				
Depression	3.1 ± 3.8 (0–25)	3.1 ± 4.3 (0–29)	2.8 ± 3.6 (0–20)	2.8 ± 3.7 (0–24)

EORTC-QLQ-C30: European Organization for Research and Treatment of Cancer Quality of Life Questionnaire: Core questionnaire, EORTC-QLQ-BR23: BReast cancer module of the EORTC-QLQ, BDI: Finnish modified version of Beck’s 13-item depression scale, QOL: Quality of Life, SD: standard deviation.

### Network structure

**Fig. 1:**
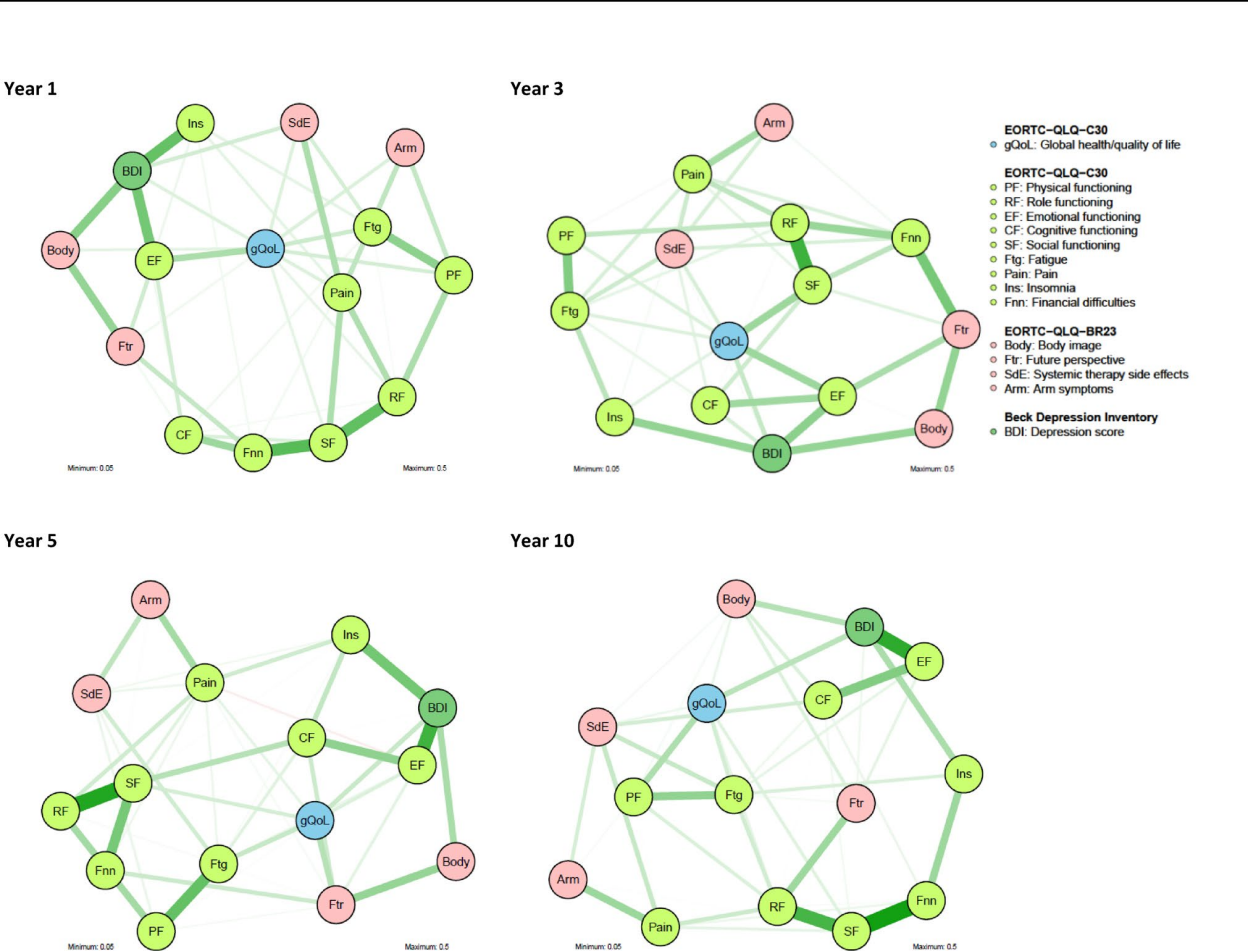
Networks constructed via graphical LASSO visualizing the regularized partial correlations between global health/QoL, symptoms, functioning and depression, measured by EORTC-QLQ C30, EORTC-QLQ B23 and BDI questionnaires, at one-, three-, five- and ten-years follow-up. *Note*. Green edges represent positive partial correlations and red edges negative ones. Thicker and more saturated edges represent stronger partial correlations. Edges with absolute weight above 0.05 are displayed. The distance between two nodes reflects the absolute edge weight between them (Fruchterman-Reingold layout). All edge weights are reported in Supplement Table 3. The depression score and the symptom scores have been reversed to follow the functioning scales interpretation, i.e., higher score indicates a lower level of symptoms and a better state of the patient. At Year 3 the node gQoL lies over the edge Ins-SF.

Figure 1 depicts the networks between gQoL, and selected functioning and symptom scales of QLQ-C30 and QLQ-B23 questionnaires and the BDI score, at one-, three-, five- and ten-years follow up, constructed on the whole dataset. Relatively dense regularized networks were constructed by EBIC glasso, having 68 (64.8%), 62 (59.0%), 69 (65.7%) and 63 (60%) nonzero edges, out of 105 possible ones, at years one, three, five, and ten, respectively. Analysis of network stability and accuracy (Supplement Table 2 and Supplement Figs. 1–4) indicates that the networks are fairly stable and accurately estimated.

The bootstrapped difference tests showed the edges social function—role function, emotional function—depression, fatigue—physical function and insomnia—depression to be significantly stronger than most (at least 50%) or many (a significant amount but less than 50%) other edges in the network at all time points. Other strong edges that were significantly higher than most or many other edges were social function-financial impact (years one and ten), cognitive function-emotional function (years five and ten), depression-body image (years one, three, and five), body image-future perspective (years one, three, and five) and financial impact-future perspective (year three). All edge weights are reported in Supplement Table 3.

In our analysis below, a data-driven threshold of 0.06 defines very weak edges. For nodes with direct connections to gQoL below this threshold, shorter indirect paths with gQoL were typically present in the networks.

Depression, emotional function, fatigue, physical function, and social function consistently connected with gQoL, though the edges emotional function–gQoL at year ten and physical function–gQoL at year five are very weak, with shorter indirect connections occurring via depression and fatigue, respectively. Role function showed a direct link with gQoL at years one and ten, but this connection was either absent or very weak at years three and five. A main indirect connection through social function existed at all time points. Future perspective associated with gQoL at year five, while at other time points, direct associations were very weak or absent, with key indirect paths through emotional function (years one and three), body image (year one), and role function (year ten). Body image had a direct connection with gQoL across all time points, though it was very weak at years three and five. Indirect paths via depression (all time points) and future perspective (year five) were of comparable or shorter length. Insomnia had no direct link to gQoL but consistently connected indirectly via depression. Cognitive function and gQoL were either unconnected or very weakly connected (year ten), with indirect associations between them via emotional function (all time points), social function (year three and five), or fatigue (year five). Financial impact’s link with gQoL occurred indirectly, primarily through social function. Systemic therapy side effects and pain were directly associated with gQoL, often with shorter indirect paths via fatigue (for systemic therapy at year five) or role and social function (for pain at years three and ten). Arm symptoms lacked a direct link to gQoL only at year three, with indirect connections of similar or shorter length through pain and/or systemic therapy side effects at years three to ten.

The indirect paths presented above are tentative. They were chosen for their relatively short length, but the analysis does not confirm a causal effect from the node of interest to gQoL via the intermediary node or exclude the possibility of shorter paths in the true network.

### Node importance

**Fig. 2:**
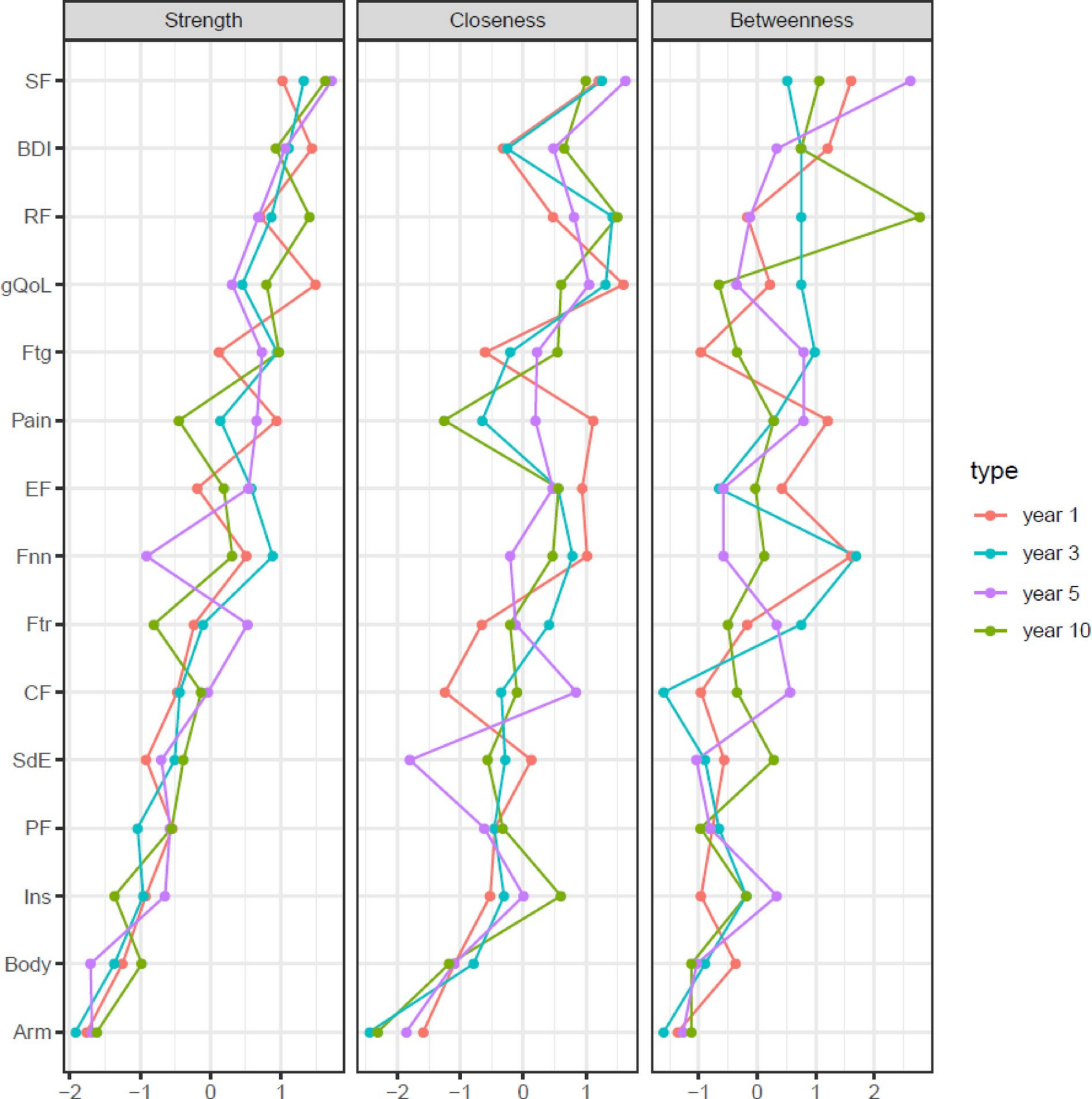
Centrality plots for graphical lasso networks at one-, three-, five- and ten-year follow-up. *Note* The strength, closeness and betweenness of each node are depicted as standardized z-scores. QoL: C30 Global Quality of Life, PF: C30 Physical functioning, RF: C30 Role functioning, SF: C30 Social functioning, CF: C30 Cognitive functioning, EF: C30 Emotional functioning, BDI: BDI Depression score, Ftg: C30 Fatigue, Ftr: BR23 Future perspective, Body: BR23 Body image, Pain: C30 Pain, SdE: BR23 Systemic therapy side effects, Ins: C30 Insomnia, Arm: BR23 Arm symptoms, Fnn: C30 Financial Difficulties.

Figure 2 presents centrality indices (standardized z-scores) over time. Stability analysis (Supplement Table 2) indicates that the order of node strength is stable and interpretable at all time points, the order of closeness is quite stable and could be interpreted cautiously at years three, five, and ten, while the order of betweenness is not stable at all. Depression and social function consistently exhibit the highest strength centrality, while fatigue (at years three, five, and ten) and role function are also important. Pain is significant only at year one, whereas gQoL also has the highest strength centrality at that time. Arm symptoms, insomnia, and body image consistently show low strength centrality over time. For closeness, role function, social function, and gQoL are key at years three, five, and ten, while pain, systemic therapy side effects, physical function, and especially arm symptoms and body image exhibit consistently low values.

### Temporal network comparison

The network comparison test found a statistically significant difference in the edge social function–insomnia between years three and five and a statistically significant decrease in strength for the node future perspective between years five and ten. The *p*-values are given in Supplement Tables 4 and 5. No statistically significant differences were found between time points for closeness (Supplement Table 6), betweenness (Supplement Table 7), network structure, or global strength (Table 2), though statistical power limitations cannot be ruled out. Spearman correlations between the edge weights of the networks at years one, three, five, and ten (rho = 0.663–0.710, Table 2) indicate high network similarity. Overall, the results suggest a high temporal stability of the networks.

**Table 2:** Results of the network comparison test and the Spearman correlation between networks.

Time points	Network comparison test		Spearman correlation	
	Network structure	Global strength	rho	*p*-value
	(*p*-value)	(*p*-value)		
Y1–Y3	0.562	0.844	0.710	< 0.001
Y1–Y5	0.367	0.796	0.697	< 0.001
Y1–Y10	0.462	0.345	0.700	< 0.001
Y3–Y5	0.750	0.857	0.663	< 0.001
Y3–Y10	0.113	0.273	0.641	< 0.001
Y5–Y10	0.396	0.249	0.661	< 0.001

## Discussion

The present study investigated how different dimensions of breast cancer patients’ QoL measured by EORTC-C30, EORTC-BR23, and BDI are connected at one-, three-, five-, and ten-years post-treatment using a network analysis method (graphical LASSO). In the network analysis we identified those factors most central to gQoL at all time points and showed how the networks developed over time.

### Network stability over time

The results suggest broad structural similarity in the networks across the ten-year follow-up period, indicating that the scales most connected with QoL stay the same even with extended time from treatments. Our earlier study from the same cohort identified scales related to fatigue and emotional health to be the most central to QoL starting from baseline^17^, and these remained still most connected with QoL throughout this 10-year follow-up. Our results suggest that the most central aspects to target for improving QoL appear to remain relatively consistent over time.

### Key areas for long-term quality of life in breast cancer survivors

The dimensions that had direct connections with gQoL at all time points studied were depression, emotional function, social function, and fatigue, which means that these are especially relevant to the subjective evaluation of QoL and/or affected by it. This type of cross-sectional analysis cannot confirm cause and effect, only point to the existence of a connection where causality might be present. The nodes with the highest standardized strength centrality at all time-points were depression, social function, fatigue, and role function, reinforcing their importance in understanding long-term QoL. Previous studies examining long-term QoL in women suffering from breast cancer support these findings, identifying impairments in emotional functioning^7^, social functioning^7,10^, role functioning^9,10^, fatigue^5,7–11^, and depression^12^ several years after treatments. The alignment between direct connections, centrality indices, and previous research underscores the importance of targeting these areas in survivorship interventions to improve long-term QoL outcomes in breast cancer survivors.

Several strong edges were observed in the network. Not surprisingly the strongest connections occurred between social function and role function, emotional function and depression, fatigue and physical function, and insomnia and depression. Visual examination of the networks suggests clustering, where the most central dimensions to QoL form tightly linked patterns. This indicates that symptoms and functions may mutually reinforce each other over time, potentially leading to self-sustaining dynamics. This observation is in line with network theory’s positing that the interaction of different symptoms can create structures that perpetuate themselves^16^.

### Addressing specific functions and symptoms in survivorship care

Earlier studies have pointed to the relationship between fatigue and mental health in breast cancer survivors^38,39^, yet no direct connections between these two domains were observed in the present study. Instead, fatigue was strongly associated with physical functioning and linked to mental health-related scales mainly via gQoL. This suggests that improving physical function could be a more direct way to target fatigue, a conclusion consistent with prior findings from the baseline and one-year follow-ups^17^. Both physical function and fatigue have been shown to respond well to exercise interventions^38,40^.

Depression and emotional functioning were strongly linked at all time points, with both showing direct connections to gQoL. However, by year ten, the association between emotional function and gQoL was primarily indirect via depression. Given depression’s strong connections with other symptoms and functions in the network, it could be argued that managing depression could have a particularly large impact on improving QoL. Previous studies have shown that depression in breast cancer survivors can be addressed through psychosocial interventions such as cognitive-behavioral therapy and mindfulness training^41,42^. Shared underlying mechanisms, like affective dysregulation and stress reactivity, could also explain the link between depression, emotional functioning, and global quality of life (gQoL)^43^. 

Social and role function capture the ability to engage in meaningful social relationships, and daily tasks and activities. Social function showed direct connections to gQoL at all time points, whereas the association between role function and gQoL was indirect via social function. This suggests that being able to perform one’s occupational and social roles can have an impact on perceived quality of life quality through its effects on social interaction. Both scales showed high standardized strength centrality at all time points, indicating that changes in either could be expected to have a broad impact on the network, making them potential targets for intervention. Psychosocial interventions aimed at increasing social engagement and supporting role performance therefore should be explored as a potential avenue for improving long-term QoL.

Cognitive function was not directly connected to gQoL or was only weakly connected at year ten. This raises the question of whether this finding is due to the high levels of physical activity maintained in this cohort^44^, given prior research suggesting that preserving physical activity during treatment supports cognitive function^45^. Notably, the connection between cognitive function and gQoL was primarily accounted for by indirect associations via emotional functioning, suggesting that cognitive difficulties may indirectly impact overall quality of life by influencing emotional well-being. However, because the networks were estimated cross-sectionally at each time point, these findings should not be interpreted as evidence of temporal invariance at the individual level or of within-person longitudinal dynamics.

Systemic therapy side-effects were still connected to perceived QoL in the later follow-ups, albeit mainly via fatigue at year five. Earlier research has shown that symptoms related to endocrine therapy can persist for years after the end of treatment^6,46^. However, it is also possible that these reported symptoms partly reflect other chronic conditions exacerbated by aging. Prolonged symptoms may also become increasingly difficult to tolerate over time, contributing to their continued impact on QoL. Further research is needed to better understand the nature of these late effects and their implications for long-term survivorship care.

An important consideration for intervention planning is the extent to which specific aspects of QoL are amenable to long-term change through behavioral or psychosocial interventions^47,48^. Other factors such as cost, feasibility, and adherence also must be considered, as long-term adherence to lifestyle interventions is often a challenge^49^. While identifying key factors influencing long-term QoL is essential, research efforts must also focus on ensuring that interventions lead to sustained improvements. In an earlier Finnish study using the international and well validated Information Needs Questionnaire (INQ) it was showed that Behavioral Escape-Avoidance coping was associated with cancer patients’ worse QoL^50^.

### Limitations

The study included a large patient sample and a follow-up period extending up to ten years, with a relatively homogeneous group in terms of cancer treatment. However, some limitations must be considered. First, due to the study design, the sample consisted primarily of patients with relatively high baseline gQoL and level of functioning, comparable to Swedish reference values in a non-clinical sample, which may cause ceiling effect and limit the generalizability^51^. Another limitation is the reliance on self-reported questionnaire data, which introduces a potential for response biases and subjective interpretations of symptom severity.

A further limitation relates to variable selection. The network was estimated only for scales that had shown at least moderate associations with gQoL at baseline and 1 year in our previous work. Although this strategy was clinically motivated and intended to focus on the most relevant QoL domains, it may have biased the network toward constructs more closely related to gQoL and may have excluded domains with weaker early but potentially indirect or delayed associations at later follow-up. The findings should therefore be interpreted as applying to the selected subset of QoL domains rather than to the full range of EORTC QLQ-C30 and BR23 scales.

The network analysis method employed here, based on partial correlations, is cross-sectional (networks were constructed separately at each wave) and cannot establish causal relationships between the included variables or capture within-person temporal dynamics. The associations identified may reflect direct effects, bidirectional influences, shared underlying processes, or semantic similarities in the questionnaire items. Likewise, scales of high centrality can either have strong influence on the network or be influenced by it, or both. Furthermore, the networks represent group-level associations, potentially masking individual variability in symptom trajectories and responses to treatment. Therefore, the broad structural similarity observed across follow-up waves should not be interpreted as evidence of temporal invariance at the individual level.

Future research should aim to replicate these findings across more heterogeneous samples, including individuals with greater health challenges at baseline. The effects of lifestyle as well as sociodemographic and clinical factors should also be evaluated. Additionally, incorporating objective measures of physical function and cognitive performance could strengthen future network analyses by reducing reliance on subjective self-reporting. Further, performing separate network analyses for different subgroups could also broaden our understanding of individual QoL trajectories. Finally, future studies could also employ dynamic approaches to examine temporal and directional relationships between quality of life (QoL) and various domains of functioning and symptoms, capturing both within-person temporal dynamics and between-person differences. For datasets with only a few waves, such as the present study, methods like cross-lagged panel models (CLPM), their improved random-intercept variant (RI-CLPM), or panel network models, including the cross-lagged panel network model (CLPN) and the panel graphical vector autoregression model (panel GVAR), may be appropriate. For intensive longitudinal datasets with many repeated measurements, multilevel vector autoregression (mlVAR) or GVAR can be applied.

## Conclusions

This study demonstrated using a network analysis (Graphical Lasso) that the factors most central to QoL remained stable across the ten-year follow-up period in the Breast cancer and Exercise (BREX) trial. Three key intervention targets were identified as most strongly linked to QoL: (1) fatigue and physical function, (2) emotional functioning and depression, and (3) social and role functioning. Future research should explore ways to support these three areas of functioning, as well as examine the long-term effects of interventions and adherence to behavioral changes.

## Supplementary Information

Below is the link to the electronic supplementary material.

Supplementary Material 1

## Data Availability

The data that support the findings of this study are available on request from the corresponding author, PPS. The data are not publicly available due to restrictions, for example, they contain information that could compromise the privacy of the study participants.
