# Supplementary material for: Network analysis of quality of life and depression in a randomized breast cancer trial with ten-years follow-up
Source: Sci Rep. 2026 May 4;16:20575. doi: 10.1038/s41598-026-51322-3 (PMC13333999; doi:10.1038/s41598-026-51322-3)
Supplement: Supplementary file 1 — Supplementary Material 1 [file 41598_2026_51322_MOESM1_ESM.docx]

**Supplement**

***Methods***

***Network’s structure stability:*** The stability of each network’s structure was evaluated using the R package bootnet, following the guidelines in Epskamp et al. (2018). Firstly, the correlation stability coefficient (CS-coefficient) was computed for edge weights and node centralities via case-dropping subset bootstrap. The CS-coefficient indicates the maximum proportion of cases that can be dropped while ensuring that the correlation between the original network and the bootstrap network remains at least 0.7 with 95% probability. The CS-coefficient ranges from 0 to 1 and should ideally be at least 0.25 for the examined aspect of the network to be deemed sufficiently stable and interpretable. Values surpassing 0.5 indicate a high level of stability (Epskamp et al., 2018). Subsequently, the sampling variability in edge-weights was explored by bootstrapping 95% confidence intervals (CIs) around them. Finally, a bootstrap analysis was utilized to identify pairs of edge weights or node strength-centralities that exhibit significant differences. 1000 bootstrapped networks were computed for each analysis.

***Temporal stability:*** A comparative analysis of the networks was conducted at years one, three, five, and ten using two approaches. Initially, the differences between the networks were assessed employing the permutation-based statistical test implemented in the R package NetworkComparisonTest (van Borkulo et al., 2016). Specifically, the edge weights, node strength centralities, global strength (the absolute sum of edge weights), and network structure (maximum absolute difference in edge weights) were examined for significant differences among the networks (van Borkulo et al., 2016). To account for the within-individual clustering in the longitudinal design, the dependent version of the test was utilized, applicable to longitudinal data of a single patient group, and 5000 permutations were performed. A significance level of .005 was considered, as the test was not corrected for multiple testing. Subsequently, the Spearman correlations between the edge weights of the networks were calculated.

**Supplement Table 1**. Baseline clinical characteristics of the study participants. Total number of patients n=364

| **Variable** | **Mean (range)** |
| --- | --- |
| Age | 52.7 (35-68) |
| **Variable** | **n (%)** |
| pT – tumour size |  |
| T1 | 209 (57.4%) |
| T2 | 132 (36.3%) |
| T3 | 18 (4.9%) |
| T4 | 3 (0.8%) |
| Tis | 1 (0.3%) |
| Tx | 1 (0.3%) |
| pN –number of axillary lymph nodes with cancer |  |
| N0 | 135 (37.1%) |
| N1 | 139 (38.2%) |
| N2 | 31 (8.5%) |
| N1mi | 39 (10.7%) |
| N3 | 8 (2.2%) |
| N0i+ | 12 (3.3%) |
| Estrogen receptor |  |
| Positive | 298 (81.9%) |
| Negative | 66 (18.1%) |
| Progesterone receptor |  |
| Positive | 242 (66.5%) |
| Negative | 122 (33.5%) |
| HER2 (human epidermal growth factor receptor 2) |  |
| Positive | 59 (16.2%) |
| Negative | 111 (30.5) |
| Unknown | 194 (53.3) |
| Breast surgery |  |
| Breast-conserving | 231 (63.5%) |
| Mastectomy | 133 (36.5%) |
| Adjuvant chemotherapy |  |
| Yes | 325 (89.3%) |
| No | 39 (10.7%) |
| Endocrine therapy |  |
| Yes | 300 (82.4%) |
| No | 64 (17.6%) |
| Radiotherapy |  |
| Yes | 281 (77.2%) |
| No | 83 (22.8%) |
| Menopause status before cancer diagnosis |  |
| Postmenopausal | 201 (55.2%) |
| Premenopausal | 163 (44.8%) |
| Trastuzumab treatment |  |
| Yes | 59 (16.2%) |
| No | 305 (83.8%) |

Abbreviations:

T1 = 1-10 mm, T2 = 11-20 mm, T3 = 21-50 mm, T4 > 51 mm, N0 = 0, N1 = 1-3, N2 = 4-9, N1Mi = micro metastases in the axillary lymph nodes, N3 > 10, or sub clavicular lymph nodes with cancer, N0i+ = isolated tumour cells in the axillary lymph nodes

***Network stability and accuracy***

Supplement Figure 1 shows the average correlation between the node centralities of the original glasso networks and the ones of the bootstrapped subsets as a function of the size of the subsets. Supplement Table 2 contains the CS coefficients for edge weights and node centralities for the glasso networks. Results indicate that the order of node strength and edge weight are stable under subsetting cases for all four time points (*CS*(cor=0.7) > 0.5). The stability of closeness is moderate for years 3, 5 and 10 (*CS*(cor=0.7) > 0.25). On the other hand, CS-coefficients for betweenness fail to reach the minimum threshold of 0.25, indicating unreliable estimates for all time points. Thus, results are interpretable for edge weights and node strength at all time points and for closeness at years 3, 5 and 10. Supplement Figure 2 reveals small to medium 95% CIs around edge weights. The results of the significant difference test show that many edges (Supplement Figure 3) and node strengths (Supplement Figure 4) significantly differ from one-another. Overall, the results indicate that the networks are fairly stable and accurately estimated and support the findings of the analysis of the network structure.

**Supplement Table 2:** Correlation stability (CS) coefficients for node centralities and edge weights for EBIC glasso networks of the whole dataset at 1-, 3-, 5- and 10-year follow up.

|  | **betweenness** | **closeness** | **edge weight** | **strength** |
| --- | --- | --- | --- | --- |
| Y1 | 0.09 | 0.12 | 0.64 | 0.60 |
| Y3 | 0.09 | 0.42 | 0.60 | 0.71 |
| Y5 | 0.09 | 0.31 | 0.64 | 0.60 |
| Y10 | 0.12 | 0.42 | 0.64 | 0.60 |

**Supplement Figure 1. Stability of centrality indices at 1-, 3-, 5- and 10-year follow up:** average correlation between the centrality indices of the original network and the ones calculated from a subsample of patients derived via case-dropping subset bootstrap. Lines indicate the means and areas indicate the range from the 2.5th quantile to the 97.5th quantile.

| **Year 1**   | **Year 3**   |
| --- | --- |
| **Year 5**   | **Year 10**   |

**Supplement Figure 2. Accuracy of the edge-weights at 1-, 3-, 5- and 10-year follow up:** bootstrapped confidence intervals of the edge-weights for the glasso networks at 1-, 3-, 5- and 10-year follow up. The red line indicates the sample value of the edge-weight, the black line the bootstrap mean and the grey area the 95% confidence intervals. Each horizontal line represents one edge of the network, ordered from the edge with the highest edge-weight to the edge with the lowest edge-weight.

| **Year 1**   | **Year 3**   |
| --- | --- |
| **Year 5**  **** | **Year 10**   |

**Supplement Figure 3. Bootstrapped difference tests (α = 0*.*05) between non-zero edge-weights in the glasso networks at 1-, 3-, 5- and 10-year follow up.** Black boxes represent pair of edges that have a significant difference in weight and gray boxes indicate pair of edges that do not have a significant difference in weight. Blue boxes correspond to edges having a positive weight (positive partial correlation between connected nodes) and red boxes correspond to edges having a negative weight (negative partial correlation between connected nodes).

The BDI depression score and the C30/BR23 symptom scores have been reversed to follow the C30/BR23 functioning scales interpretation, i.e. higher score indicates a lower level of symptoms and a better state of the patient. Abbreviations: **gQoL**: C30 Global Quality of Life, **PF**: C30 Physical functioning, **RF**: C30 Role functioning, **SF**: C30 Social functioning, **CF**: C30 Cognitive functioning, **EF**: C30 Emotional functioning, **BDI**: BDI Depression score, **Ftg**: C30 Fatigue, **Fnn**: C30 Financial Difficulties, **Ftr**: BR23 Future perspective, **Body**: BR23 Body image, **Pain**: C30 Pain, **SdE**: BR23 Systemic therapy side effects, **Ins**: C30 Insomnia, **Arm**: BR23 Arm symptoms.

| **Year 1** | **Year 3** |
| --- | --- |
| **Year 5** | **Year 10** |

**Supplement Figure 4. Bootstrapped difference tests (α = 0*.*05) between node strength in the glasso networks at 1-, 3-, 5- and 10-year follow up.** Black boxes represent pair of nodes that significantly differ from one another in terms of in strength and gray boxes indicate pair of nodes that do not significantly differ from one another. White boxes show the value of the node strength.

The BDI depression score and the C30/BR23 symptom scores have been reversed to follow the C30/BR23 functioning scales interpretation, i.e. higher score indicates a lower level of symptoms and a better state of the patient. Abbreviations: **gQoL**: C30 Global Quality of Life, **PF**: C30 Physical functioning, **RF**: C30 Role functioning, **SF**: C30 Social functioning, **CF**: C30 Cognitive functioning, **EF**: C30 Emotional functioning, **BDI**: BDI Depression score, **Ftg**: C30 Fatigue, **Fnn**: C30 Financial Difficulties, **Ftr**: BR23 Future perspective, **Body**: BR23 Body image, **Pain**: C30 Pain, **SdE**: BR23 Systemic therapy side effects, **Ins**: C30 Insomnia, **Arm**: BR23 Arm symptoms.

| **Year 1**   | **Year 3**   |
| --- | --- |
| **Year 5**   | **Year 10**   |

***Correlation coefficients***

**Supplement Table 3 S1.2:** Correlation coefficients of EBIC glasso networks at 1-, 3-, 5- and 10-year follow up. The BDI depression score and the C30/BR23 symptom scores have been reversed to follow the C30/BR23 functioning scales interpretation, i.e. higher score indicates a lower level of symptoms and a better state of the patient. Abbreviations: **gQoL**: C30 Global Quality of Life, **PF**: C30 Physical functioning, **RF**: C30 Role functioning, **SF**: C30 Social functioning, **CF**: C30 Cognitive functioning, **EF**: C30 Emotional functioning, **BDI**: BDI Depression score, **Fatigue**: C30 Fatigue, **Financial**: C30 Financial Difficulties, **Future**: BR23 Future perspective, **Body**: BR23 Body image, **Pain**: C30 Pain, **SideEffects**: BR23 Systemic therapy side effects, **Insomnia**: C30 Insomnia, **Arm**: BR23 Arm symptoms.

*Edges with absolute weight below 0.05 are not displayed in Fig1.

***Network Comparison Test***

**Supplement Table 4.** p-values per edge from the permutation test concerning differences in edges weights between the networks at 1-, 3-, 5- and 10-year follow up.

| **Node 1** | **Node 2** | **Y1-Y3** | **Y1-Y5** | **Y1-Y10** | **Y3-Y5** | **Y3-Y10** | **Y5-Y10** |
| --- | --- | --- | --- | --- | --- | --- | --- |
| gQoL | PF | 0.87 | 0.368 | 0.391 | 0.388 | 0.249 | 0.064 |
| gQoL | RF | 0.126 | 0.528 | 0.999 | 0.427 | 0.174 | 0.541 |
| PF | RF | 0.599 | 0.87 | 0.265 | 0.538 | 0.576 | 0.256 |
| gQoL | EF | 0.68 | 0.176 | 0.023 | 0.083 | 0.022 | 0.343 |
| PF | EF | 1 | 1 | 1 | 1 | 1 | 1 |
| RF | EF | 0.083 | 1 | 1 | 0.108 | 0.064 | 1 |
| gQoL | CF | 1 | 1 | 0.133 | 1 | 0.517 | 0.117 |
| PF | CF | 0.152 | 1 | 1 | 0.218 | 0.764 | 1 |
| RF | CF | 0.944 | 0.687 | 0.893 | 0.691 | 0.946 | 0.793 |
| EF | CF | 0.149 | 0.09 | 0.022 | 0.687 | 0.407 | 0.71 |
| gQoL | SF | 0.114 | 0.764 | 0.92 | 0.204 | 0.17 | 0.83 |
| PF | SF | 0.177 | 0.984 | 0.557 | 0.197 | 1 | 0.604 |
| RF | SF | 0.63 | 0.283 | 0.662 | 0.629 | 0.899 | 0.56 |
| EF | SF | 1 | 1 | 1 | 1 | 1 | 1 |
| CF | SF | 0.84 | 0.553 | 0.721 | 0.668 | 0.529 | 0.301 |
| gQoL | Ftg | 0.679 | 0.861 | 0.879 | 0.532 | 0.813 | 0.737 |
| PF | Ftg | 0.752 | 0.291 | 0.918 | 0.42 | 0.682 | 0.24 |
| RF | Ftg | 0.699 | 0.994 | 0.523 | 0.694 | 0.737 | 0.575 |
| EF | Ftg | 0.469 | 0.221 | 0.164 | 0.624 | 0.559 | 0.899 |
| CF | Ftg | 0.875 | 0.718 | 0.975 | 0.8 | 0.898 | 0.683 |
| SF | Ftg | 1 | 1 | 0.577 | 1 | 0.643 | 0.679 |
| gQoL | Pain | 0.187 | 0.818 | 0.182 | 0.344 | 0.838 | 0.261 |
| PF | Pain | 0.957 | 0.388 | 0.671 | 0.422 | 0.619 | 0.185 |
| RF | Pain | 0.796 | 0.592 | 0.612 | 0.798 | 0.844 | 0.953 |
| EF | Pain | 0.406 | 0.63 | 0.372 | 0.128 | 1 | 0.121 |
| CF | Pain | 0.354 | 0.136 | 0.269 | 1 | 1 | 1 |
| SF | Pain | 0.42 | 0.419 | 0.452 | 0.947 | 0.983 | 0.961 |
| Ftg | Pain | 0.45 | 0.82 | 0.621 | 0.58 | 0.233 | 0.416 |
| gQoL | Ins | 1 | 1 | 1 | 1 | 1 | 1 |
| PF | Ins | 1 | 1 | 1 | 1 | 1 | 1 |
| RF | Ins | 0.158 | 0.258 | 0.551 | 1 | 1 | 1 |
| EF | Ins | 0.796 | 1 | 1 | 0.357 | 0.382 | 1 |
| CF | Ins | 0.413 | 0.035 | 1 | 0.165 | 0.527 | 0.029 |
| SF | Ins | 0.229 | 0.207 | 1 | 0.005 | 0.14 | 0.269 |
| Ftg | Ins | 0.271 | 0.635 | 0.779 | 0.127 | 0.456 | 0.424 |
| Pain | Ins | 0.732 | 0.624 | 0.29 | 0.363 | 0.478 | 0.116 |
| gQoL | Fnn | 1 | 1 | 1 | 1 | 1 | 1 |
| PF | Fnn | 0.668 | 0.686 | 0.747 | 0.515 | 0.498 | 1 |
| RF | Fnn | 0.295 | 0.668 | 0.996 | 0.461 | 0.285 | 0.655 |
| EF | Fnn | 0.009 | 0.859 | 0.063 | 0.014 | 0.235 | 0.174 |
| CF | Fnn | 0.098 | 0.018 | 0.318 | 0.429 | 0.577 | 0.259 |
| SF | Fnn | 0.157 | 0.691 | 0.358 | 0.339 | 0.035 | 0.16 |
| Ftg | Fnn | 0.029 | 0.155 | 0.054 | 1 | 1 | 1 |
| Pain | Fnn | 0.496 | 0.694 | 0.672 | 0.797 | 0.832 | 0.997 |
| Ins | Fnn | 0.523 | 0.337 | 0.265 | 1 | 0.121 | 0.069 |
| gQoL | Body | 0.488 | 0.157 | 0.869 | 0.414 | 0.709 | 0.264 |
| PF | Body | 0.508 | 0.639 | 0.129 | 0.371 | 0.047 | 0.478 |
| RF | Body | 1 | 0.331 | 1 | 0.077 | 1 | 0.291 |
| EF | Body | 0.756 | 0.151 | 0.383 | 0.483 | 0.767 | 1 |
| CF | Body | 0.645 | 0.698 | 0.13 | 0.387 | 0.235 | 0.061 |
| SF | Body | 1 | 0.233 | 1 | 0.804 | 1 | 0.237 |
| Ftg | Body | 1 | 1 | 1 | 1 | 1 | 1 |
| Pain | Body | 1 | 1 | 1 | 1 | 1 | 1 |
| Ins | Body | 0.766 | 0.39 | 0.744 | 0.259 | 0.977 | 0.078 |
| Fnn | Body | 1 | 0.644 | 0.209 | 0.504 | 0.254 | 0.168 |
| gQoL | Ftr | 0.333 | 0.078 | 0.942 | 0.006 | 0.493 | 0.061 |
| PF | Ftr | 1 | 0.035 | 0.145 | 0.047 | 0.214 | 0.693 |
| RF | Ftr | 0.389 | 0.751 | 0.007 | 0.261 | 0.008 | 0.062 |
| EF | Ftr | 0.367 | 0.636 | 0.641 | 0.187 | 0.221 | 0.957 |
| CF | Ftr | 0.262 | 0.432 | 0.571 | 0.056 | 0.656 | 0.138 |
| SF | Ftr | 0.145 | 1 | 0.509 | 0.346 | 0.602 | 0.755 |
| Ftg | Ftr | 0.651 | 0.912 | 0.81 | 0.576 | 0.496 | 0.896 |
| Pain | Ftr | 0.459 | 0.47 | 0.447 | 0.197 | 1 | 0.283 |
| Ins | Ftr | 0.67 | 0.324 | 0.18 | 0.537 | 0.405 | 1 |
| Fnn | Ftr | 0.26 | 0.939 | 0.151 | 0.259 | 0.014 | 0.094 |
| Body | Ftr | 0.943 | 0.985 | 0.059 | 0.937 | 0.082 | 0.057 |
| gQoL | SdE | 0.905 | 0.259 | 0.664 | 0.41 | 0.684 | 0.499 |
| PF | SdE | 0.854 | 0.43 | 0.949 | 0.321 | 0.936 | 0.343 |
| RF | SdE | 0.261 | 0.568 | 0.053 | 1 | 1 | 1 |
| EF | SdE | 1 | 0.379 | 0.438 | 0.111 | 0.226 | 0.936 |
| CF | SdE | 0.324 | 0.719 | 0.126 | 0.17 | 0.574 | 0.081 |
| SF | SdE | 1 | 0.098 | 1 | 0.025 | 1 | 0.023 |
| Ftg | SdE | 0.808 | 0.891 | 0.701 | 0.924 | 0.895 | 0.797 |
| Pain | SdE | 0.372 | 0.113 | 0.522 | 0.367 | 0.853 | 0.333 |
| Ins | SdE | 0.522 | 0.562 | 0.945 | 0.151 | 0.363 | 0.566 |
| Fnn | SdE | 0.049 | 1 | 1 | 0.059 | 0.031 | 1 |
| Body | SdE | 1 | 0.849 | 0.29 | 0.76 | 0.243 | 0.369 |
| Ftr | SdE | 0.845 | 0.667 | 0.902 | 0.584 | 1 | 0.367 |
| gQoL | Arm | 0.042 | 0.392 | 0.542 | 0.38 | 0.37 | 0.858 |
| PF | Arm | 0.198 | 0.018 | 0.104 | 0.283 | 0.718 | 0.543 |
| RF | Arm | 1 | 1 | 1 | 1 | 1 | 1 |
| EF | Arm | 1 | 0.109 | 1 | 0.175 | 1 | 0.053 |
| CF | Arm | 1 | 1 | 1 | 1 | 1 | 1 |
| SF | Arm | 1 | 0.12 | 0.083 | 0.065 | 0.046 | 0.777 |
| Ftg | Arm | 0.155 | 1 | 0.14 | 0.703 | 0.739 | 0.396 |
| Pain | Arm | 0.52 | 0.53 | 0.429 | 0.997 | 0.815 | 0.817 |
| Ins | Arm | 1 | 1 | 1 | 1 | 1 | 1 |
| Fnn | Arm | 0.634 | 0.735 | 0.823 | 0.869 | 0.447 | 0.541 |
| Body | Arm | 1 | 0.83 | 1 | 0.568 | 1 | 0.48 |
| Ftr | Arm | 0.089 | 0.212 | 0.195 | 1 | 1 | 1 |
| SdE | Arm | 0.204 | 0.083 | 0.185 | 0.548 | 0.976 | 0.533 |
| gQoL | BDI | 0.47 | 0.507 | 0.332 | 0.972 | 0.712 | 0.677 |
| PF | BDI | 1 | 1 | 1 | 1 | 1 | 1 |
| RF | BDI | 1 | 1 | 1 | 1 | 1 | 1 |
| EF | BDI | 0.565 | 0.127 | 0.054 | 0.057 | 0.017 | 0.537 |
| CF | BDI | 0.528 | 0.759 | 0.202 | 0.379 | 0.112 | 0.377 |
| SF | BDI | 0.066 | 0.471 | 0.246 | 1 | 1 | 1 |
| Ftg | BDI | 0.113 | 0.166 | 1 | 0.8 | 0.355 | 0.34 |
| Pain | BDI | 0.515 | 0.573 | 1 | 0.777 | 0.133 | 0.072 |
| Ins | BDI | 0.237 | 0.665 | 0.067 | 0.383 | 0.559 | 0.062 |
| Fnn | BDI | 0.68 | 1 | 1 | 0.474 | 0.514 | 1 |
| Body | BDI | 0.703 | 0.339 | 0.226 | 0.609 | 0.452 | 0.733 |
| Ftr | BDI | 1 | 1 | 0.338 | 1 | 0.178 | 0.278 |
| SdE | BDI | 0.372 | 0.207 | 0.193 | 0.613 | 0.702 | 0.921 |
| Arm | BDI | 0.47 | 1 | 1 | 0.539 | 0.172 | 1 |

**Supplement Table 5.** p-values per node from the permutation test concerning differences in strength centralities between the networks at 1-, 3-, 5- and 10-year follow up.

| **Node** | **Y1-Y3** | **Y1-Y5** | **Y1-Y10** | **Y3-Y5** | **Y3-Y10** | **Y5-Y10** |
| --- | --- | --- | --- | --- | --- | --- |
| gQoL | 0.040 | 0.039 | 0.099 | 0.822 | 0.764 | 0.557 |
| PF | 0.175 | 0.911 | 0.715 | 0.182 | 0.438 | 0.779 |
| RF | 0.692 | 0.867 | 0.376 | 0.845 | 0.618 | 0.452 |
| EF | 0.193 | 0.283 | 0.684 | 0.973 | 0.357 | 0.305 |
| CF | 0.931 | 0.322 | 0.699 | 0.328 | 0.766 | 0.554 |
| SF | 0.655 | 0.317 | 0.585 | 0.383 | 0.915 | 0.507 |
| Ftg | 0.034 | 0.093 | 0.240 | 0.748 | 0.761 | 0.952 |
| Pain | 0.283 | 0.885 | 0.018 | 0.475 | 0.184 | 0.045 |
| Ins | 0.906 | 0.681 | 0.289 | 0.495 | 0.257 | 0.236 |
| Fnn | 0.662 | 0.086 | 0.738 | 0.037 | 0.479 | 0.198 |
| Body | 0.693 | 0.228 | 0.843 | 0.269 | 0.542 | 0.100 |
| Ftr | 0.724 | 0.078 | 0.281 | 0.117 | 0.095 | 0.003 |
| SdE | 0.409 | 0.632 | 0.438 | 0.605 | 0.926 | 0.716 |
| Arm | 0.629 | 0.843 | 0.912 | 0.612 | 0.628 | 0.929 |
| BDI | 0.762 | 0.826 | 0.407 | 0.944 | 0.521 | 0.326 |

**Supplement Table 6.** p-values per node from the permutation test concerning differences in closeness centralities between the networks at 1-, 3-, 5- and 10-year follow up.

| **Node** | **Y1-Y3** | **Y1-Y5** | **Y1-Y10** | **Y3-Y5** | **Y3-Y10** | **Y5-Y10** |
| --- | --- | --- | --- | --- | --- | --- |
| gQoL | 0.416 | 0.900 | 0.151 | 0.340 | 0.114 | 0.411 |
| PF | 0.936 | 0.560 | 0.662 | 0.503 | 0.608 | 0.982 |
| RF | 0.109 | 0.733 | 0.590 | 0.254 | 0.365 | 0.838 |
| EF | 0.729 | 0.797 | 0.477 | 0.480 | 0.269 | 0.772 |
| CF | 0.473 | 0.144 | 0.771 | 0.473 | 0.684 | 0.262 |
| SF | 0.388 | 0.553 | 0.775 | 0.867 | 0.279 | 0.394 |
| Ftg | 0.578 | 0.565 | 0.596 | 0.972 | 0.993 | 0.970 |
| Pain | 0.220 | 0.608 | 0.010 | 0.630 | 0.120 | 0.088 |
| Ins | 0.773 | 0.871 | 0.736 | 0.942 | 0.895 | 0.856 |
| Fnn | 0.641 | 0.387 | 0.587 | 0.169 | 0.391 | 0.811 |
| Body | 0.882 | 0.518 | 0.261 | 0.396 | 0.197 | 0.732 |
| Ftr | 0.237 | 0.870 | 0.851 | 0.265 | 0.163 | 0.710 |
| SdE | 0.911 | 0.043 | 0.236 | 0.007 | 0.227 | 0.163 |
| Arm | 0.088 | 0.171 | 0.034 | 0.555 | 0.836 | 0.448 |
| BDI | 0.839 | 0.580 | 0.746 | 0.699 | 0.875 | 0.814 |

**Supplement Table 7.** p-values per node from the permutation test concerning differences in betweenness centralities between the networks at 1-, 3-, 5- and 10-year follow up.

| **Node** | **Y1-Y3** | **Y1-Y5** | **Y1-Y10** | **Y3-Y5** | **Y3-Y10** | **Y5-Y10** |
| --- | --- | --- | --- | --- | --- | --- |
| gQoL | 0.818 | 0.705 | 0.414 | 0.520 | 0.377 | 0.805 |
| PF | 0.900 | 1 | 0.685 | 0.894 | 0.429 | 0.797 |
| RF | 0.647 | 1 | 0.038 | 0.685 | 0.121 | 0.031 |
| EF | 0.499 | 0.483 | 0.711 | 1 | 0.585 | 0.616 |
| CF | 0.577 | 0.243 | 0.585 | 0.085 | 0.377 | 0.633 |
| SF | 0.562 | 0.760 | 0.944 | 0.301 | 0.628 | 0.686 |
| Ftg | 0.056 | 0.111 | 0.535 | 0.925 | 0.331 | 0.507 |
| Pain | 0.480 | 0.727 | 0.480 | 0.833 | 0.930 | 0.927 |
| Ins | 0.437 | 0.309 | 0.543 | 0.789 | 1 | 0.760 |
| Fnn | 0.943 | 0.109 | 0.497 | 0.171 | 0.527 | 0.563 |
| Body | 0.626 | 0.313 | 0.121 | 0.896 | 0.308 | 0.648 |
| Ftr | 0.336 | 0.748 | 0.678 | 0.717 | 0.169 | 0.384 |
| SdE | 0.847 | 0.469 | 0.207 | 0.896 | 0.289 | 0.089 |
| Arm | 1 | 0.308 | 1 | 0.485 | 1 | 0.565 |
| BDI | 0.659 | 0.424 | 0.940 | 0.740 | 0.783 | 0.526 |
